# Transcriptome analysis of adenomyosis eutopic endometrium reveals molecular mechanisms involved in adenomyosis-related implantation failure and pregnancy disorders

**DOI:** 10.1186/s12958-023-01182-7

**Published:** 2024-01-09

**Authors:** Elena Juárez-Barber, Ana Corachán, María Cristina Carbajo-García, Amparo Faus, Carmen Vidal, Juan Giles, Antonio Pellicer, Irene Cervelló, Hortensia Ferrero

**Affiliations:** 1 Fundación IVI, Instituto de Investigación Sanitaria La Fe, Valencia, 46026 Spain; 2 Department of Pediatrics, Obstetrics and Gynecology, Universidad de Valencia, Valencia, 46010 Spain; 3 IVI-RMA Valencia, Valencia, 46015 Spain; 4 IVI-RMA Rome, Rome, 00197 Italy

**Keywords:** Adenomyosis, Endometrial organoids, RNA-sequencing, Implantation failure, Pregnancy loss, Infertility

## Abstract

**Background:**

Women with adenomyosis are characterized by having defective decidualization, impaired endometrial receptivity and/or embryo-maternal communication, and implantation failure. However, the molecular mechanisms underlying adenomyosis-related infertility remain unknown, mainly because of the restricted accessibility and the difficult preservation of endometrial tissue in vitro. We have recently shown that adenomyosis patient-derived endometrial organoids, maintain disease-specific features while differentiated into mid-secretory and gestational endometrial phase, overcoming these research barriers and providing a robust platform to study adenomyosis pathogenesis and the associated molecular dysregulation related to implantation and pregnancy disorders. For this reason, we aim to characterize the dysregulated mechanisms in the mid-secretory and gestational endometrium of patients with adenomyosis by RNA-sequencing.

**Methods:**

Endometrial organoids were derived from endometrial biopsies collected in the proliferative phase of women with adenomyosis (ADENO) or healthy oocyte donors (CONTROL) (n = 15/group) and differentiated into mid-secretory (-SECorg) and gestational (-GESTorg) phases in vitro. Following RNA-sequencing, the significantly differentially expressed genes (DEGs) (FDR < 0.05) were identified and selected for subsequent functional enrichment analysis and QIAGEN Ingenuity Pathway Analysis (IPA). Statistical differences in gene expression were evaluated with the Student’s t-test or Wilcoxon test.

**Results:**

We identified 1,430 DEGs in ADENO-SECorg and 1,999 DEGs in ADENO-GESTorg. In ADENO-SECorg, upregulated genes included *OLFM1*, *FXYD5*, and *RUNX2*, which are involved in impaired endometrial receptivity and implantation failure, while downregulated genes included *RRM2, SOSTDC1*, and *CHAC2* implicated in recurrent implantation failure. In ADENO-GESTorg, upregulated *CXCL14* and *CYP24A1* and downregulated *PGR* were related to pregnancy loss. IPA predicted a significant inhibition of ID1 signaling, histamine degradation, and activation of HMGB1 and Senescence pathways, which are related to implantation failure. Alternatively, IPA predicted an inhibition of D-myo-inositol biosynthesis and VEGF signaling, and upregulation of Rho pathway, which are related to pregnancy loss and preeclampsia.

**Conclusions:**

Identifying dysregulated molecular mechanisms in mid-secretory and gestational endometrium of adenomyosis women contributes to the understanding of adenomyosis-related implantation failure and/or pregnancy disorders revealing potential therapeutic targets. Following experimental validation of our transcriptomic and *in silico* findings, our differentiated adenomyosis patient-derived organoids have the potential to provide a reliable platform for drug discovery, development, and personalized drug screening for affected patients.

**Supplementary Information:**

The online version contains supplementary material available at 10.1186/s12958-023-01182-7.

## Introduction

Adenomyosis is a benign uterine disease, defined as an infiltration of the endometrial glands and stroma into the myometrium [1]. It affects approximately 35% of reproductive-aged women [2], although the prevalence can vary depending on the study population, diagnostic methods, and geographic location [3]. Women with adenomyosis present abnormal uterine bleeding, chronic pelvic pain, dysmenorrhea, dyspareunia, and infertility [4], driving them to seek assisted reproductive technologies [5]. However, in vitro fertilization efficacy for these patients remains highly controversial, with some studies reporting lower implantation rates but no effect on miscarriage rates [6, 7], and others describing frequent miscarriages without any adverse effects on implantation or pregnancy rates [8, 9]. Nevertheless, meta-analyses concluded that women with adenomyosis had higher miscarriage rates, lower implantation, pregnancy, and live birth rates compared to healthy patients [10–13], suggesting adenomyosis may impair embryo implantation and early pregnancy [13]. In this regard, understanding the underlying molecular mechanisms involved in adenomyosis pathogenesis is essential for managing adenomyosis-related infertility.

Defective decidualization [14], impaired endometrial receptivity [15], and/or embryo-maternal communication [16], and implantation failure [17] have been described in women with adenomyosis. However, the molecular mechanisms underlying these infertility-related alterations in adenomyosis women remain unknown, mainly due to the limited availability and difficult maintenance of the eutopic and ectopic endometrial tissues in vitro. As embryo implantation occurs in the endometrial mid-secretory phase [18], and events related to the embryo-maternal communication and early pregnancy stages happen in the endometrial gestational phase [19], deciphering the transcriptome of these endometrial phases in women with adenomyosis will represent a step forward in understanding the dysregulation that contributes to adenomyosis-associated infertility.

Organoids have emerged as a three dimensional (3D) in vitro platform capable of reproducing the phenotypes of native tissues remaining genetically stable in long-term culture [20]. Endometrial organoids have been developed from healthy and diseased endometrium, mimicking endometriosis [21], endometrial cancer [21], and adenomyosis [22], among other conditions. Notably, patient-derived adenomyosis endometrial organoids differentiated into mid-secretory and gestational phase phenotypes maintain disease-specific traits, overcoming the aforementioned research barriers and providing a reliable model to study adenomyosis pathogenesis and associated molecular dysregulation related to implantation and pregnancy disorders. In this regard, our adenomyosis organoids model allowed us to describe microRNAs contained in extracellular vesicles (EVs) secreted by these adenomyosis secretory and gestational organoids, involved in impaired embryo implantation and pregnancy disorders related with this disease [23]. However, there is not any study describing molecular mechanisms deregulated in eutopic endometrium in secretory and gestational phase from women with adenomyosis. Therefore, the aim of our study was to analyze the transcriptome of adenomyosis-derived endometrial organoids in the mid-secretory and gestational phases, to characterize the molecular mechanisms involved in adenomyosis-related infertility.

## Materials and methods

### Study design

Endometrial organoids were derived from the eutopic endometrium of women with (n = 15) or without adenomyosis (control; n = 15) and further differentiated into mid-secretory and gestational endometrial phases by supplementation with ovarian and pregnancy hormones [22], respectively. RNA was extracted from mid-secretory and gestational adenomyosis and control endometrial organoids for RNA-sequencing (RNA-seq) (Supplemental Fig. 1).

### Patients and endometrial biopsies

Endometrial biopsies were obtained from patients (18 ≤ 45 years old; BMI ≤ 28 kg/m^2^) with and without adenomyosis, at the IVI Valencia Clinic (Table 1). Patients with any other suspected or diagnosed uterine pathologies were excluded. Control women were healthy egg donors with standard uterine volume, no evidence of adenomyotic lesions, and free of other gynecological pathologies and medication during previous three months.

**Table 1:** Basic demographic parameters. Age is measured in years and BMI in kg/m^2^

PATIENT	AGE	BMI	RACE	PARITY	TYPE OF ADENOMYOSIS
ADENO 1	42	26.40	White	IF2, G4, M1, LB3	Focal
ADENO 2	38	26.18	White	-	Focal
ADENO 3	40	19.83	White	-	Focal
ADENO 4	39	21.30	White	IF2, G2, M2	Focal
ADENO 5	37	22.32	NR	IF2	Diffuse
ADENO 6	42	22.90	White	IF2	Cystic and Diffuse
ADENO 7	41	22.15	White	IF1, G1, M1	Diffuse
ADENO 8	42	25.26	White	-	Diffuse
ADENO 9	44	26.37	Hispanic	-	Focal
ADENO 10	37	28.00	White	IF1	NR
ADENO 11	36	19.94	Hispanic	IF1, G4, M4	Diffuse
ADENO 12	34	23.14	NR	G2, M2	Focal
ADENO 13	42	21.87	White	IF2, G2, M2	Focal
ADENO 14	38	28.00	NR	IF1, G2, M2	Focal
ADENO 15	34	23.53	White	-	Focal
CONTROL 1	22	24.37	White	-	-
CONTROL 2	22	21.95	White	-	-
CONTROL 3	19	23.87	White	-	-
CONTROL 4	28	23.74	White	-	-
CONTROL 5	23	20.55	Hispanic	-	-
CONTROL 6	29	21.87	White	G3, 2 M, LB1	-
CONTROL 7	24	22.13	Hispanic	-	-
CONTROL 8	21	21.30	NR	-	-
CONTROL 9	23	22.15	NR	-	-
CONTROL 10	25	20.18	NR	-	-
CONTROL 11	28	23.11	NR	-	-
CONTROL 12	22	19.96	White	-	-
CONTROL 13	27	22.12	White	G1, LB1	-
CONTROL 14	31	25.52	NR	-	-
CONTROL 15	28	25.64	White	G1, M1	-

IF: implantation failure; G: gestation; M: miscarriage; LB: live-birth; NR: non referred

### Diagnosis of adenomyosis

All patients were examined by transvaginal ultrasound. Adenomyosis was diagnosed in patients presenting a heterogeneous myometrium and a diffused endometrial border. Diffuse adenomyosis was diagnosed with a globally enlarged asymmetric uterus, hypoechoic striae, and areas with small cysts in the intramyometrial region, while focal adenomyosis was diagnosed by isolated intramyometrial clusters surrounded by areas of normal myometrium and altered vascularity [24, 25]. In all cases adenomyosis was confirmed by hysteroscopic evaluation of the endometrial cavity.

### Establishment and differentiation of adenomyosis endometrial organoids

The adenomyosis and control endometrial organoids were derived from eutopic endometrium and differentiated into the mid-secretory and gestational phases modelling native endometrial tissue and disease-specific traits, which showed in vivo glandular epithelial phenotype (pan-cytokeratin, Mucin-1 [Muc-1], Periodic acid Schiff [PAS] staining, Laminin, and Ki67; assessed by immunostaining) and secretory and gestational features (α-tubulin, SRY-Box Transcription Factor 9 [SOX9], Secreted Phosphoprotein 1 [*SPP1*], Progestagen Associated Endometrial Protein [*PAEP*], LIF Interleukin 6 Family Cytokine [*LIF*], and Hydroxysteroid 17-Beta Dehydrogenase 2 [*17βHSD2*] expression and SPP1 secretion, assessed by immunostaining and quantitative real-time PCR (qRT-PCR)), as we previously described [22]. Immunohistochemistry of adenomyosis organoids showed higher expression of Transforming Growth Factor Beta 2 [TGFβ-2] and SMAD Family Member 3 [SMAD3] and increased gene expression of *SPP1*, *PAEP*, *LIF*, and *17βHSD2* by means of qRT-PCR [22]. Briefly, for mid-secretory phase differentiation, adenomyosis (ADENO-SECorg) and control organoids (CONTROL-SECorg) were treated with 10 nM estradiol (E2; Sigma-Aldrich, St. Louis, MO, USA, E4389), 1 µM progesterone (P4; Sigma-Aldrich, P7556) and 1 µM 8-bromoadenosine 3′,5′-cyclic monophosphate sodium salt (cAMP; Sigma-Aldrich, B7880). For gestational phase differentiation, adenomyosis (ADENO-GESTorg) and control organoids (CONTROL-GESTorg) were treated with 10 nM E2, 1 µM P4, 1 µM cAMP, with an additional 20 ng/mL prolactin (PRL; Peprotech, Cranbury, NJ, USA, 100-07) and 20 ng/mL human placental lactogen (hPL; R&D, Minneapolis, MN, USA, 5757-PL).

### Library construction and RNA-sequencing

Total RNA was extracted from the ADENO-SECorg, CONTROL-SECorg, ADENO-GESTorg and CONTROL-GESTorg groups (n = 15/group) using the RNeasy Mini Kit (Qiagen, Germantown, MD, USA, 74,104) according to the manufacturer’s protocol, and quantified with a Qubit 3 Fluorometer (Invitrogen, Waltham, MA, USA).

Next, cDNA libraries were generated employing the TruSeq Stranded mRNA Library Prep (Illumina, San Diego, CA, USA, 20,020,595) and TruSeq RNA CD Index Plate (Illumina, 20,019,792) according to manufacturer’s instructions. The quality and concentration of the libraries was assessed with the Agilent Technologies 2100 (Agilent Technologies, Santa Clara, CA, USA, G2939BA). Paired-end sequencing (2 × 75 bp) was performed on Illumina’s NextSeq 550 NGS platform.

### Pre-processing, quality control and normalization

RNA-seq data libraries were processed within R computing environment (v 4.1.1). Library quality was analyzed with FastQC software [26]. Low-quality sequences (e.g., from one CONTROL-SECorg and two CONTROL-GESTorg samples) were removed with bbduk software [27]. Sequencing samples yielded an average of 14.1 million reads per sample. RNA-seq reads were aligned with the GRCh38 version of the human genome using subread software [28]. Read counts were normalized using the geometric median ratio method for each mRNA, using the DESeq2 R package. All raw sequencing data are available through the Gene Expression Omnibus (GEO) under accession number GSE244236.

### Differentially expressed genes and functional enrichment analysis

Differential expression analysis (DEA) was carried out with the DESeq2 package to identify the differentially expressed genes (DEGs) between: (i) ADENO-SECorg versus CONTROL-SECorg; and (ii) ADENO-GESTorg versus CONTROL-GESTorg. Differentially expressed genes (DEGs) were considered significant when the P-value adjusted by false discovery rate (FDR) < 0.05. Gene ontology functional enrichment analysis and KEGG pathway analysis were performed by gene set enrichment analysis (GSEA) implemented in clusterProfiler [29]. Finally, the QIAGEN Ingenuity Pathway Analysis (IPA) was used to analyze the dysregulated pathways in both comparisons.

### Validation

To corroborate RNA-seq data, we selected DEGs implicated in dysregulated pathways described in ADENO-SECorg and evaluated their gene expression by qRT-PCR using Power-Up SYBR Green (Thermo Fisher Scientific, USA) on a StepOnePlus Real-Time PCR System (Applied Biosystems, USA). The selected genes included Aldehyde Dehydrogenase 1 Family Member A1 (ALDH1A1), Aldehyde Dehydrogenase 9 Family Member A1 (ALDH9A1), Monoamine Oxidase B (MAOB), Lysine Acetyltransferase 2B (KAT2B), Poly (ADP-Ribose) Polymerase 1 (PARP1), Forkhead Box O3 (FOXO3), Superoxide Dismutase 2 (SOD2) and Sequestosome 1 (SQSTM1). Relative gene expression levels were determined by the ∆∆Ct method and normalized to β-actin (ACTB) housekeeping gene expression. Fold change was calculated using the CONTROL-SECorg as the reference group.

### Statistical analysis

All statistical analyses of omics data were carried out in R (v 4.1.1). Graphics were generated using the R core package, gplots, ggplot2, or GraphPad Prism 8.0. Statistical differences in gene expression were evaluated with the Student’s t-test or Wilcoxon test in GraphPad Prism 8.0. In all cases, P < 0.05 was considered statistically significant.

## Results

### Global transcriptomic behaviour of adenomyosis patient-derived organoids

**Fig. 1:**
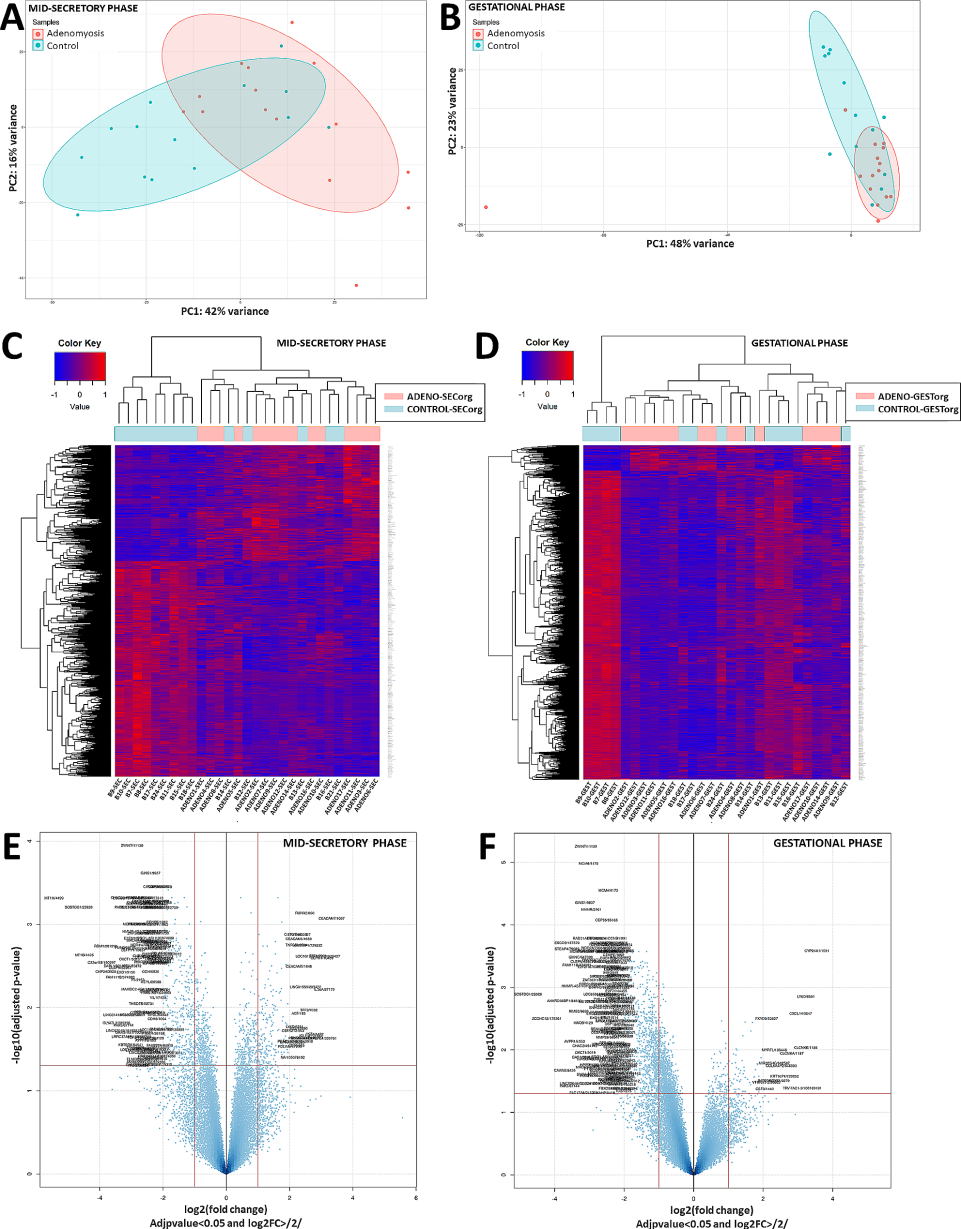
Global transcriptomic behavior of secretory and gestational endometrial organoids from patients with adenomyosis compared to healthy oocyte donors. Principal component analyses (**A**-**B**), heatmaps representing the fold-enrichment score of genes after unsupervised clustering (**C**-**D**), and volcano plots of the significantly differentially expressed genes (adjusted p value < 0.05 and log2FC>|2|; **E**-**F**) between ADENO-SECorg (red; n = 15) and CONTROL-SECorg (blue; n = 14), or ADENO-GESTorg (red; n = 15) and CONTROL-GESTorg (blue; n = 13)

Principal Component Analysis (PCA) revealed distinct transcriptomic behaviour between the ADENO-SECorg and CONTRO-SECorg samples (Fig. 1A) and between the ADENO-GESTorg and CONTROL-GESTorg samples (Fig. 1B). In corroboration, the hierarchically-clustered heatmaps of the significant mRNAs (FDR < 0.05) showed different expression patterns between ADENO-SECorg and CONTROL-SECorg (Fig. 1C) and between ADENO-GESTorg and CONTROL-GESTorg (Fig. 1D).

### Differential gene expression of mid-secretory phase adenomyosis endometrial organoids

**Fig. 2:**
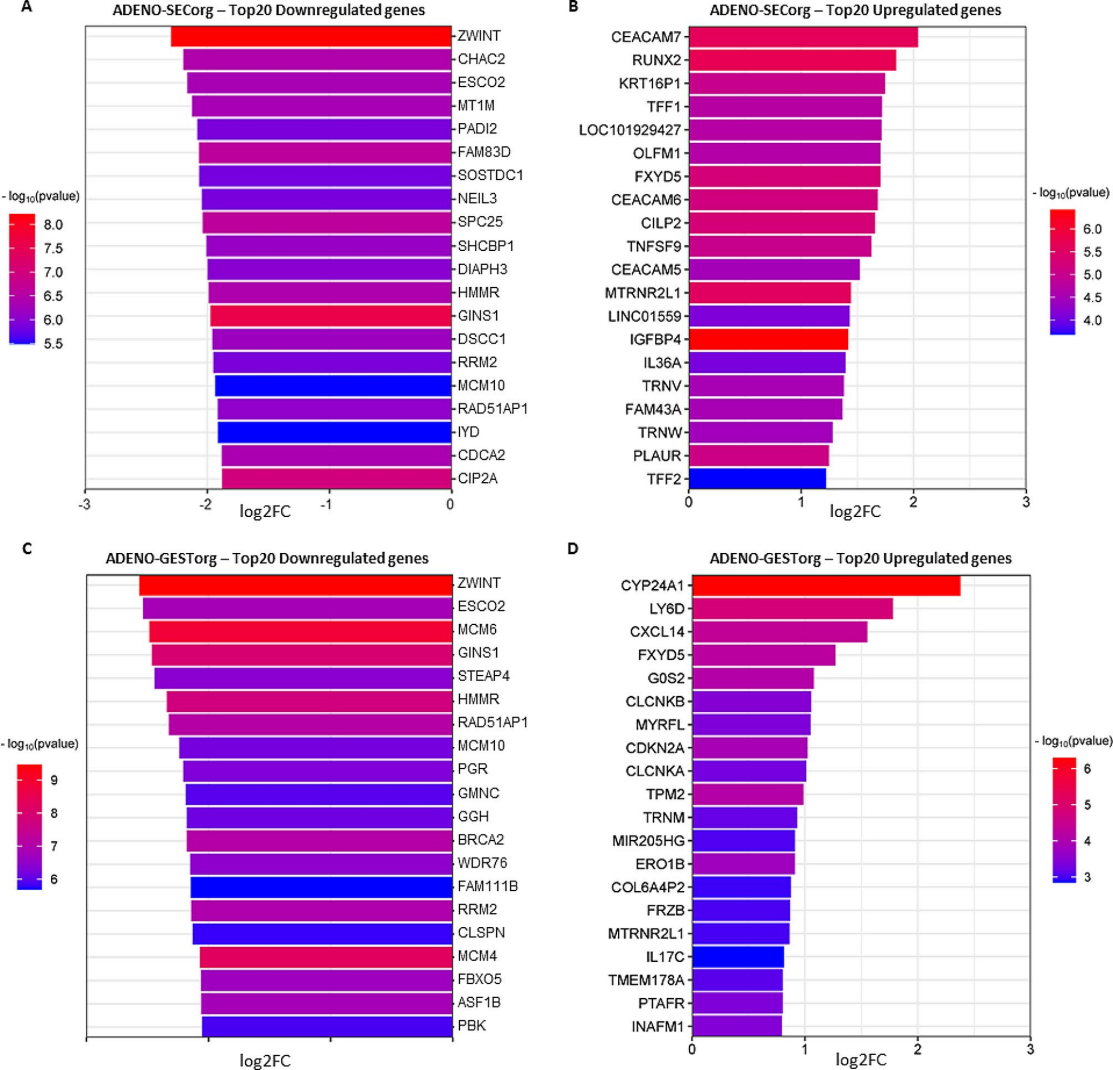
Top 20 significantly differentially expressed genes between adenomyosis and control patient-derived endometrial organoids in mid-secretory and gestational phases. (**A**) Downregulated and (**B**) upregulated genes in ADENO-SECorg compared to CONTROL-SECorg. (**C**) Downregulated and (**D**) upregulated genes in ADENO-GESTorg compared to CONTROL-GESTorg. Statistical significance of the presented genes was established with adjusted p value < 0.05

DEA identified 1,430 DEGs (500 up- and 930 downregulated; FDR < 0.05) between the ADENO-SECorg and CONTRO-SECorg in the mid-secretory phase (Fig. 1E). Among the top 20 downregulated DEGs selected for subsequent analysis (Fig. 2A), we highlight ChaC Glutathione Specific Gamma-Glutamylcyclotransferase 2 [*CHAC2*], Metallothionein 1M [*MT1M*], Sclerostin Domain Containing 1 [*SOSTDC1*], and Ribonucleotide Reductase Regulatory Subunit M2 [*RRM2*] (log2 Fold change [FC] = -2.20, -2.13, -2.07, and − 1.95, respectively) based on their possible implication in recurrent implantation failure (RIF). Alternatively, among the top 20 upregulated DEGs (Fig. 2B) we point out RUNX Family Transcription Factor 2 [*RUNX2*], Olfactomedin 1 [*OLFM1*], FXYD Domain Containing Ion Transport Regulator 5 [*FXYD5*], and MT-RNR2 Like 1 [*MTRNR2L1*] (log2FC = 1.84, 1.70, 1.70, and 1.44, respectively) due to their involvement in impaired endometrial receptivity and embryo implantation.

### Differential gene expression of gestational phase adenomyosis endometrial organoids

DEA identified 1,999 DEGs (153 up- and 1,846 downregulated; FDR < 0.05 and) between the ADENO-GESTorg and CONTROL-GESTorg in the gestational phase (Fig. 1F). Among the top 20 downregulated DEGs (Fig. 2C), we highlight ZW10 Interacting Kinetochore Protein (*ZWINT*), Establishment Of Sister Chromatid Cohesion N-Acetyltransferase 2 (*ESCO2*), Minichromosome Maintenance Complex Component 6 (*MCM6*), progesterone receptor (*PGR*) and Minichromosome Maintenance Complex Component 4 (*MCM4*) (log2FC = -2.57, -2.53, -2.48, -2.21, and − 2.07, respectively) based on their possible associations with recurrent pregnancy loss (RPL) and preeclampsia. From the top 20 upregulated DEGs (Fig. 2D), we note Cytochrome P450 Family 24 Subfamily A Member 1 (*CYP24A1*), *C-X-C Motif Chemokine Ligand 14* (*CXCL14*), Cyclin Dependent Kinase Inhibitor 2 A (*CDKN2A*), Chloride Voltage-Gated Channel Ka (*CLCNKA*) and Platelet Activating Factor Receptor (*PTAFR*) (log2FC = 2.38, 1.55, 1.02, 1.01, and 0.80, respectively) due to their implication in spontaneous miscarriage, trophoblast outgrowth and invasion inhibition, and gestational diabetes mellitus.

### Functional implications of adenomyosis in the mid-secretory phase endometrium

**Fig. 3:**
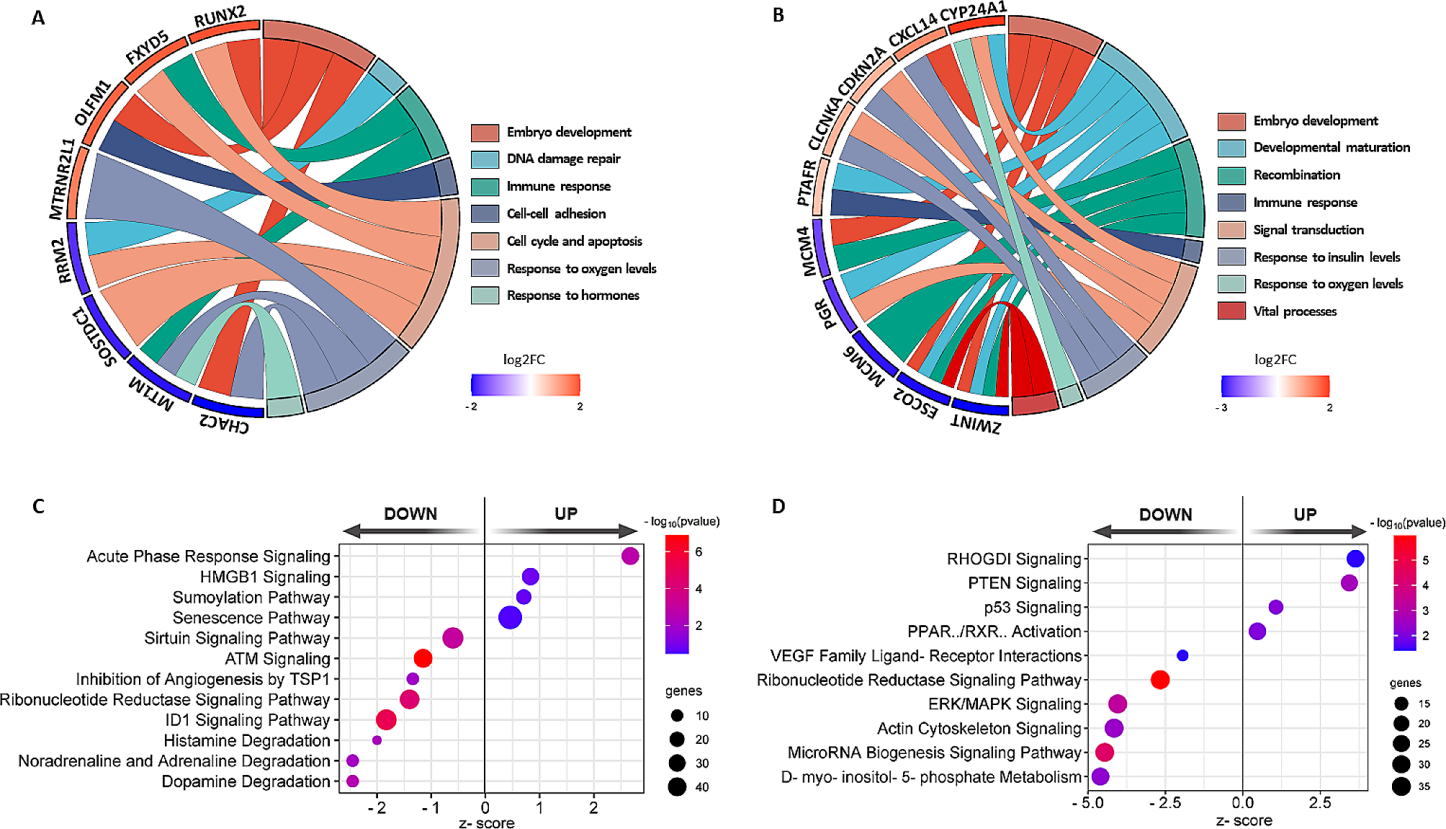
Functional enrichment analysis and canonical pathways predicted to be affected by Ingenuity Pathway Analysis (IPA). Functional implications of relevant significantly downregulated and upregulated genes in (**A**) ADENO-SECorg with respect to CONTROL-SECorg or (**B**) ADENO-GESTorg with respect to CONTROL-GESTorg. Differential expression of the genes is showed in a box under the gene in blue (downregulated) and red (upregulated) by means of log2FC scale. Downregulated and upregulated canonical pathways predicted by IPA and deemed relevant for adenomyosis pathogenesis and associated-infertility in (**C**) ADENO-SECorg and (**D**) ADENO-GESTorg. DEGs, differentially expressed genes

GO enrichment analysis identified 176 dysregulated biological processes in ADENO-SECorg (Supplemental Table 1). These processes were assigned to different functional groups, such as oocyte and embryo development, DNA damage repair, response to oxygen levels and hormones, immune response, cell-cell adhesion, cell cycle and apoptosis, aligning with the described functions of the mid-secretory phase DEGs we emphasized herein (Fig. 3A). On the other hand, KEGG pathway analysis revealed nine dysregulated pathways related to the cell cycle, mismatch repair, homologous recombination, cellular senescence, estrogen and progesterone signaling, inflammation cascades and different types of viral infection, among others (Supplemental Table 1).

### Functional implications of adenomyosis in the gestational phase endometrium

GO analysis revealed 356 dysregulated biological processes in ADENO-GESTorg (Supplemental Table 2). Among the corresponding functional groups, embryo development, vital processes, developmental maturation, recombination, response to oxygen levels, radiation, insulin and stimulus, signal transduction and immune response, all stood out for their possible involvement in pregnancy disorders and corroborated the previously published associations of the gestational phase DEGs we featured (Fig. 3B). Further, KEGG pathway analysis revealed 39 dysregulated pathways, related to homologous recombination, mismatch repair, apoptosis and p53 signaling, different types of cancer, viral infection, diabetic complications and inflammation signaling cascades, among others (Supplemental Table 2).

### Adenomyosis-related dysregulated pathways in the mid-secretory phase endometrium

QIAGEN IPA predicted 36 downregulated and 21 upregulated canonical pathways in the mid-secretory endometrium of women with adenomyosis, compared to controls (Supplemental Table 3). Among the relevant downregulated pathways in the mid-secretory endometrium, we distinguished the degradation of noradrenaline and adrenaline (z-score=-2.4), dopamine (z-score=-2.4), and histamine (z-score=-2.0), along with the inhibitor of DNA binding 1 (ID1) signaling pathway (z-score=-1.8), ribonucleotide reductase signaling pathway (z-score=-1.4), inhibition of angiogenesis by thrombospondin 1 (TSP1; z-score=-1.3), ATM signaling (z-score=-1.1), and sirtuin signaling pathway (z-score=-0.60), (Fig. 3C). Alternatively, among the upregulated pathways, we emphasize acute phase response signaling (z-score = 2.7), high mobility group box 1 (HMGB1) signaling (z-score = 0.8), sumoylation (z-score = 0.7) and senescence pathways (z-score = 0.5) (Fig. 3C).

### Adenomyosis-related dysregulated pathways in the gestational phase endometrium

QIAGEN IPA analysis predicted 141 downregulated and 14 upregulated canonical pathways in the gestational phase endometrium of women with adenomyosis, compared to controls (Supplemental Table 4). Among the ones relevant for adenomyosis pathogenesis and infertility (Fig. 3D), D-myo-inositol-5-phosphate metabolism (z-score=-4.6), and signaling pathways for microRNA biogenesis (z-score=-4.5), the actin cytoskeleton (z-score=-4.2), extracellular signal-regulated kinase (ERK)/mitogen-activated protein kinase (MAPK) (z-score=-4.0) and ribonucleotide reductase (z-score=-2.7), together with vascular endothelial growth factor (VEGF) family ligand-receptor interactions (z-score=-1.9), were predicted as downregulated. Meanwhile, the predicted upregulated pathways included those for Rho GDP dissociation inhibitor (RHOGDI; z-score = 3.6), phosphatase and tensin homolog (PTEN; z-score = 3.4), p53 (z-score = 1.1) and peroxisome proliferator activated receptor alpha (PPARα)/retinoid X receptor alpha (RXRα) activation (z-score = 0.5).

### Validation of differential gene expression in adenomyosis organoids

**Fig. 4:**
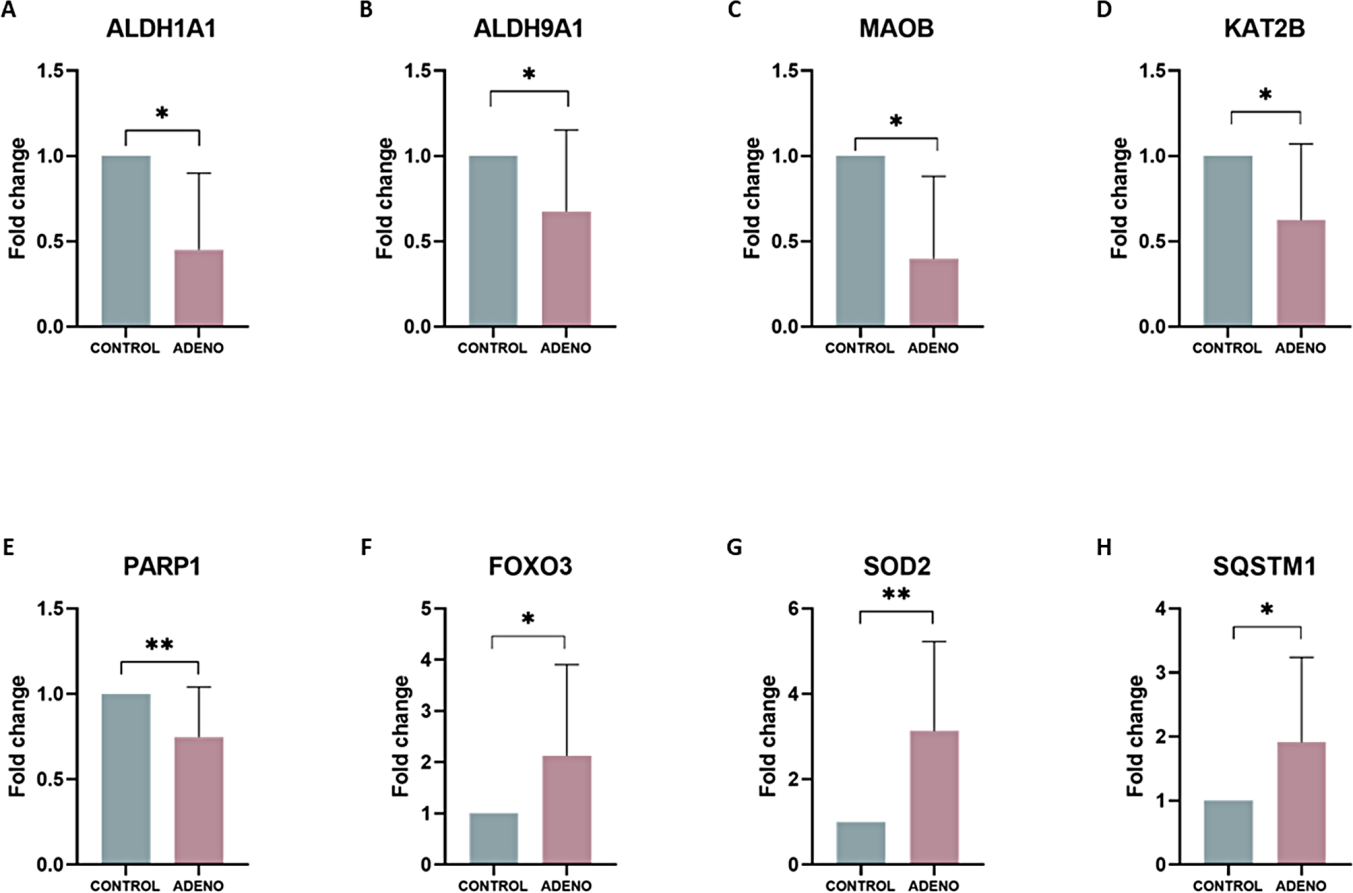
Validation of RNAseq results in ADENO-SECorg. Gene expression of (**A**) ALDH1A1, (**B**) ALDH9A1, (**C**) MAOB, (**D**) KAT2B, (**E**) PARP1, (**F**) FOXO3, (**G**) SOD2 and (**H**) SQSTM1 was validated in ADENO-SECorg by qRT-PCR. * p < 0.05; ** p < 0.005

To validate RNA-sequencing results, eight DEGs were selected among the genes involved in the dysregulated pathways Histamine degradation, Dopamine degradation, Noradrenaline and Adrenaline degradation and Senescence in ADENO-SECorg. qRT-PCR results corroborated the differential gene expression pattern observed in ADENO-SECorg by RNA-seq compared to CONTROL-SECorg (Fig. 4). Specifically, ALDH1A1 (fold change = 0.235; p = 0.023), ALDH9A1 (fold change = 0.675; p = 0.035), MAOB (fold change = 0.136; p = 0.023), KAT2B (fold change = 0.624; p = 0.012), PARP1 (fold change = 0.746; p = 0.009), FOXO3 (fold change = 1.820; p = 0.011), SOD2 (fold change = 3.130; p = 0.003), SQSTM1 (fold change = 1.912; p = 0.034).

## Discussion

Women with adenomyosis are characterized by impaired implantation and a higher number of miscarriages [10–13], thus, being able to study the dysregulated pathways and their putative causes in the endometrium, when these events occur, is crucial to improve fertility care for affected patients. Conventionally, the study of the endometrium in mid-secretory (implantation [30]) and gestational (early pregnancy [31]) phases was restricted by the difficulty of accessing and culturing the endometrium in these phases. However, the recent generation and differentiation of endometrial organoids overcomes these research barriers [20] and facilitates the study of specific endometrial disorders [21]. Going one step further, in this study, we performed next-generation sequencing of adenomyosis patient-derived organoids to identify the dysregulated genes and pathways in the eutopic secretory and gestational phase endometrium that may be responsible for the implantation failure and miscarriages experienced by affected women.

In ADENO-SECorg, we found *CHAC2, MT1M, SOSTDC1* and *RRM2* as significantly downregulated DEGs. *CHAC2* has a pivotal role in the neutralization of reactive oxygen species, being necessary for the maintenance of human embryonic stem cell self-renewal [32]. *MT1M* is critical for regulating oxidative stress, inflammation and hormone signaling in term and preterm labor [33]. *SOSTDC1*, was found expressed in the uterine glandular epithelial cells of the receptive rat endometrium, and thus, may be involved in the onset of endometrial receptivity [34], while *RRM2* expression was downregulated in the RIF endometrium, compared to fertile controls [35]. Based on this evidence, our findings suggest that the downregulation of these DEGs in the mid-secretory endometrium of women with adenomyosis advance the knowledge of adenomyosis and contributes to the endometrial dysfunction that impedes embryo implantation. Our findings indicate that adenomyosis-related infertility is also a product of the significant upregulation of certain DEGs, such as *RUNX2*, increased in the endometrium of infertile women with endometriosis [36]. *OLFM1*, related with a non-receptive endometrium and negatively regulates embryo attachment [37]; *FXYD5*, which drives the epithelial-to-mesenchymal transition [38] and promoted chronic inflammatory responses [39]; and *MTRNR2L1*, which was enhanced under hypoxic conditions in women with complicated pregnancies [40]. Based on the transcriptomic findings of the adenomyotic mid-secretory phase endometrium, IPA predicted the dysregulation of several pathways, that corresponded with those previously associated with poor reproductive outcomes. Particularly, downregulated histamine degradation was associated with pregnancy complications, such as diabetes, miscarriage, and trophoblastic disorders [41]; Sirtuin deficiency impaired embryo invasion and decidualization [42]; ATM-deficient dams had lower implantation rates [43]; and excessive noradrenaline inhibited decidualization, embryo, and fetal development in mice [44]. Further, impaired decidualization may be caused by aberrant stromal cell differentiation, mediated by downregulated ID1 expression, [45] stromal cell apoptosis, induced by the N-acyl dopamine family [46], or attenuation of ribonucleotide reductase signaling, which impeded decidualization and implantation in mice [35]. Finally, significant repression of TSP1 mRNA expression was linked to unexplained recurrent spontaneous abortion (URSA) [47]. On the other hand, IPA predicted mid-secretory phase adenomyosis etiologies may also include the upregulation of senescence pathway, as was observed in the peri-implantation endometrium and RPL [48]; hypersumoylation, since hyposumoylation was associated with a proper decidualization [49]; the premature activation of acute phase response signaling, which may interrupt early pregnancy [50]; and overactive HMGB1 signaling, which was related to the reduced adhesion ability of epithelial cells in patients with RIF [51] and at the maternal-fetal interface of URSA patients [52], as it was also previously described as deregulated in the endometrial tissue of adenomyosis women [53]. Findings from this study were corroborated by the validation in endometrial organoids of expression levels of DEG involved in these pathways, supporting that the dysregulated pathways in the mid-secretory endometrium of women with adenomyosis contribute to the disruption of endometrial receptivity and/or defective decidualization, resulting in these women failing to achieve implantation, and ultimately, pregnancy.

In ADENO-GESTorg, we focused on the downregulation of *ZWINT, ESCO2, MCM4/6*, and *PGR* because of their roles in pregnancy-related processes. The knockdown of ZWINT1 was related to a high incidence of aneuploidy, leading to miscarriage, infertility, and newborn disorders [54]. Interestingly, elevated aneuploidy rates were also observed in ESCO2-mutant embryos [55]. Correct DNA replication requires the proper functioning of MCM family members, including MCM6 [56]. Indeed, MCM4 dysregulation causes genomic instability, and increases lethality of murine embryos [57]. Alternatively, dysregulated PGR expression was related to severe preeclampsia [58] and predisposition to RPL [59]. Among the upregulated genes, elevated CYP24A1 was observed in spontaneous miscarriage [60] and preeclamptic placentas [61]; CXCL14 is implicated in insulin [62] and inhibited trophoblast attachment and outgrowth, disrupting the establishment of pregnancy [63]; and CDKN2A and CLCNKA were respectively associated with gestational diabetes [64] and IGF-1 deficiency [65], while PTAFR induced preterm delivery in mice [66]. Taken together, the contributions of these dysregulated genes showcase the complexity of adenomyosis pathogenesis.

Based on the findings presented herein, we emphasize several putative causes for the pregnancy disorders in patients with adenomyosis. Particularly, the downregulated D-myo-inositol-5-phosphate metabolism may decrease oocyte and embryo quality [67]; the reduced VEGF family ligand-receptor interactions may restrict the trophoblasts’ hypoxia adaptation [68]; limited actin cytoskeleton signaling may impede the polymerization essential for trophoblast invasion and tube formation during placental development [69]; attenuated microRNA biogenesis (mediated by DICER and DROSHA ribonucleases) during the endometrial receptivity phase may lead to implantation failure [70]; and repressed ERK/MAPK signaling may directly lead to embryonic lethality, as observed with the placental malformations due to the loss of Map2k1 function in mice [71]. Given the reproductive impact of the biological processes involving these pathways, their downregulation is proposed as a potential contributor to the many miscarriages suffered by women with adenomyosis. Interestingly, several pathways predicted to be affected by gestational phase adenomyosis have been related to preeclampsia, including upregulated PPARα/RXRα activation, which negatively regulated trophoblast invasion and led to recurrent miscarriage [72]; excessive p53 signaling [73]; along with enhanced RHOGDI and PTEN, which also inhibited trophoblast invasion [74, 75].

To our knowledge, this is the first transcriptomic study of adenomyosis patient-derived endometrial organoids differentiated into mid-secretory and gestational phase. Although these in vitro models faithfully recapitulated the native microenvironment in which the events related to implantation and early pregnancy respectively occur, additional in vivo studies are required to validate the DEGs and predicted pathways we identified as altered in the eutopic endometrium of women with adenomyosis. Moreover, endometrial organoids only contain epithelial cells and the complexity of interactions present in the native tissues may not be fully reflected in this model. Therefore, further studies including stromal or immune system cells would be necessary to validate and to translate our findings to the clinical practice. Nevertheless, it is important to highlight the importance of endometrial epithelial cells in the implantation and pregnancy processes because they are the first maternal contact for an implanting embryo and thereby, our organoid model could define new biomarkers of adenomyosis pathogenesis and related infertility.

## Conclusions

Dysregulated molecular mechanisms involved in defective decidualization, disrupted endometrial receptivity and impaired embryo implantation were identified in the mid-secretory phase endometrium of women with adenomyosis, whereas dysregulated molecular mechanisms associated with inhibition of trophoblast outgrowth and invasion, impaired embryo development, pregnancy loss, preeclampsia and placental defects were observed in gestational phase endometrium of women with adenomyosis. These findings represent potential therapeutic targets that can be exploited to develop pharmacological treatments, and ultimately, reduce the risk of adenomyosis-related infertility.

Our differentiated patient-derived adenomyosis organoids, together with the transcriptomic findings presented herein, can be used to develop and test targeted pre-conception therapies in vitro/ex vivo. Further, these pathological endometrial organoids can be used as personalized drug screening tools, to predict patient-specific drug efficacy in vitro prior to clinical administration.

### Electronic supplementary material

Below is the link to the electronic supplementary material.

**Supplementary Material 1: Supplementary Figure 1.** Experimental design. Created with BioRender.com

**Supplementary Material 2: Supplementary Table 1.** Functional enrichment and KEGG pathway analysis of ADENO-SECorg

**Supplementary Material 3: Supplementary Table 2.** Functional enrichment and KEGG pathway analysis of ADENO-GESTorg

**Supplementary Material 4: Supplementary Table 3.** Canonical pathways predicted to be dysregulated in ADENO-SECorg

**Supplementary Material 5: Supplementary Table 4.** Canonical pathways predicted to be dysregulated in ADENO-GESTorg

## Data Availability

All data generated or analyzed during this study are included in this published article and its supplementary information files. All raw sequencing data are available through the GEO under accession number GSE244236.

## Abbreviations

GESTorg: Gestational phase derived organoids
SECorg: Mid-secretory phase derived organoids
*17βHSD2*: Hydroxysteroid 17-Beta Dehydrogenase 2
ACTB: β-actin
ADENO: Adenomyosis
ALDH1A1: Aldehyde Dehydrogenase 1 Family Member A1
ALDH9A1: Aldehyde Dehydrogenase 9 Family Member A1
cAMP: 1 µM 8-bromoadenosine 3′,5′-cyclic monophosphate sodium salt
CDKN2A: Cyclin Dependent Kinase Inhibitor 2 A
*CHAC2*: ChaC Glutathione Specific Gamma-Glutamylcyclotransferase 2
*CLCNKA*: Chloride Voltage-Gated Channel Ka
CONTROL: Healthy oocyte donors
*CYP24A1*: Cytochrome P450 Family 24 Subfamily A Member 1
*CXCL14*: C-X-C Motif Chemokine Ligand 14
DEA: Differential expression analysis
DEGs: Differentially expressed genes
E2: Estradiol
ERK: Extracellular signal-regulated kinase
*ESCO2*: Establishment Of Sister Chromatid Cohesion N-Acetyltransferase 2
EVs: Extracellular vesicles
FC: Fold change
FDR: False discovery rate
*FOXO3*: Forkhead Box O3
*FXYD5*: FXYD Domain Containing Ion Transport Regulator 5
GEO: Gene expression omnibus
GSEA: Gene set enrichment analysis
*HMGB1*: High mobility group box 1
hPL: Human Placental Lactogen
ID1: Inhibitor of DNA binding 1
IPA: QIAGEN Ingenuity Pathway Analysis
*KAT2B*: Lysine Acetyltransferase 2B
*LIF*: LIF Interleukin 6 Family Cytokine
*MAOB*: Monoamine Oxidase B
*MAPK*: Mitogen-activated protein kinase
*MCM4*: Minichromosome Maintenance Complex Component 4
*MCM6*: Minichromosome Maintenance Complex Component 6
*MT1M*: Metallothionein 1 M
*MTRNR2L1*: MT-RNR2 Like 1
Muc-1: Mucin-1
*OLFM1*: Olfactomedin 1
P4: Progesterone
*PAEP*: Progestagen Associated Endometrial Protein
*PARP1*: Poly (ADP-Ribose) Polymerase 1
PAS: Periodic acid Schiff
PCA: Principal Component Analysis
*PGR*: Progesterone receptor
*PPARα*: Peroxisome proliferator activated receptor alpha
PRL: Prolactin
*PTAFR*: Platelet Activating Factor Receptor
*PTEN*: Phosphatase and tensin homolog
qRT-PCR: Quantitative real-time PCR
*RHOGDI*: Rho GDP dissociation inhibitor
RIF: Recurrent implantation failure
RNA-seq: RNA-sequencing
RPL: Recurrent pregnancy loss
*RRM2*: Ribonucleotide Reductase Regulatory Subunit M2
*RUNX2*: RUNX Family Transcription Factor 2
*RXRα*: Retinoid X receptor alpha
SMAD3: SMAD Family Member 3
*SOD2*: Superoxide Dismutase 2
*SOSTDC1*: Sclerostin Domain Containing 1
SOX9: SRY-Box Transcription Factor 9
*SPP1*: Secreted Phosphoprotein 1
SQSTM1: Sequestosome 1
*TGFβ*-2: Transforming Growth Factor Beta 2
*TSP1*: Thrombospondin 1
URSA: Unexplained recurrent spontaneous abortion
*VEGF*: Vascular endothelial growth factor
*ZWINT*: ZW10 Interacting Kinetochore Protein
