# Supplementary figures and images for: Transcriptome analysis of adenomyosis eutopic endometrium reveals molecular mechanisms involved in adenomyosis-related implantation failure and pregnancy disorders
Source: Reprod Biol Endocrinol. 2024 Jan 9;22:10. doi: 10.1186/s12958-023-01182-7 (PMC10775471; doi:10.1186/s12958-023-01182-7)

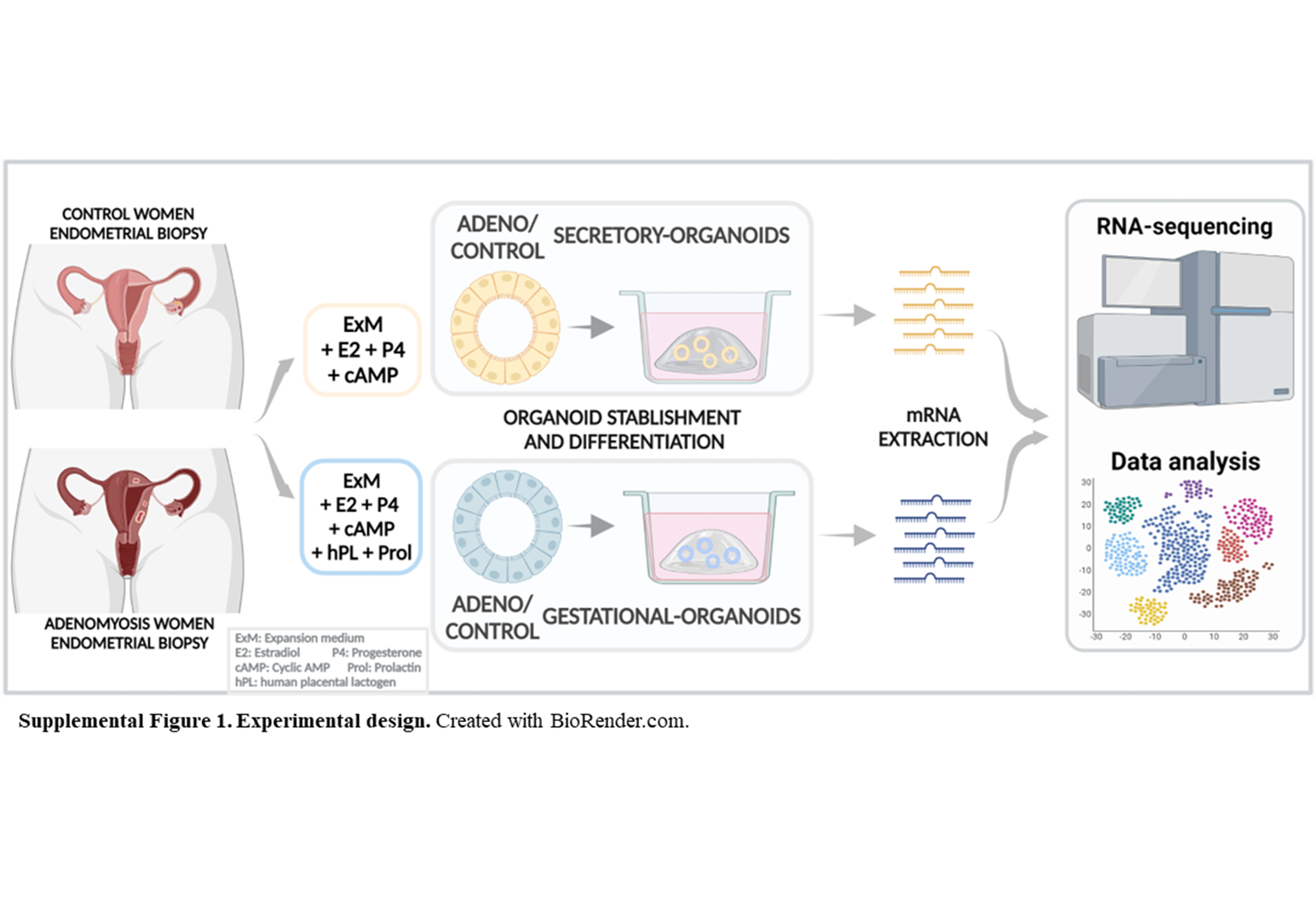

Supplement: Supplementary file 1 — Supplementary Material 1: Supplementary Figure 1. Experimental design. Created with BioRender.com [file 12958_2023_1182_MOESM1_ESM.tif]
